# The contribution of probiotics for the double-edge effect of cefazolin on postoperative neurocognitive disorders by rebalancing the gut microbiota

**DOI:** 10.3389/fnins.2023.1156453

**Published:** 2023-04-27

**Authors:** Tianyao Zhang, Xiaochu Wu, Bin Liu, Han Huang, Cheng Zhou, Peng Liang

**Affiliations:** ^1^Department of Anesthesiology, West China Hospital of Sichuan University, Chengdu, China; ^2^Department of Anesthesiology, The First Affiliated Hospital of Chengdu Medical College, Chengdu, China; ^3^National Clinical Research Center for Geriatrics, West China Hospital, Sichuan University, Chengdu, China; ^4^Laboratory of Anesthesia and Critical Care Medicine, Translational Neuroscience Center, West China Hospital of Sichuan University, Chengdu, China; ^5^Department of Anesthesiology and Key Laboratory of Birth Defects and Related Diseases of Women and Children, Ministry of Education, West China Second Hospital of Sichuan University, Chengdu, Sichuan, China; ^6^Day Surgery Center, General Practice Medical Center, West China Hospital, Sichuan University and the Research Units of West China (2018RU012), Chinese Academy of Medical Sciences, Chengdu, China

**Keywords:** cefazolin, gut dysbacteria, neuroinflammation, NLRP3 inflammasome, perioperative neurocognitive disorders, probiotics

## Abstract

**Introduction:**

Emerging data suggest that perioperative gut dysbiosis is prevalent and may be associated with postoperative neurocognitive disorders (PND). Antibiotics and probiotics are key factors influencing the microbiota. Many antibiotics have anti-microorganisms and direct anti-inflammatory properties, which may have cognitive repercussions. NLRP3 inflammasome activation has been reported to be involved with cognitive deficits. This study aimed to determine the effect and mechanism of probiotics on neurocognitive problems associated with perioperative gut dysbiosis by the NLRP3 pathway.

**Methods:**

In a randomized, controlled trial, adult male Kunming mice undergoing surgery were administered cefazolin, FOS + probiotics, CY-09, or a placebo in four distinct experimental cohorts. Fear conditioning (FC) tests evaluate learning and memory. Following FC tests to evaluate inflammatory response (IR) and the permeability of barrier systems, the hippocampus and colon were extracted, and feces were collected for 16 s rRNA.

**Results:**

One week after surgery, surgery/anesthesia decreased the frozen behavior. Cefazolin attenuated this declination but aggravated postoperative freezing behavior 3 weeks after surgery. Probiotics ameliorated surgery/anesthesia-induced memory deficits and perioperative cefazolin-induced postoperative memory deficits 3 weeks after surgery. NLRP3, caspase-1, Interleukin-1β (IL-1β), and Interleukin-18 (IL-18) levels were increased 1 week after the hippocampus and colon surgery, which were attenuated by CY-09 and probiotics, respectively.

**Discussion:**

Probiotics could correct dysbacteria and IR caused by surgery/anesthesia stress and cefazolin alone. These findings imply that probiotics are an efficient and effective way of maintaining the balance of gut microbiota, which may reduce NLRP3-related inflammation and alleviate PND.

## Background

Cognitive functions are directly tied to the gut microbiome. Intestinal microbiota can bi-directionally regulate brain function through the circulation (Loman et al., 2019), vagus (Zhang et al., 2020; Wang S. et al., 2021), and endocranium systems, which are collectively regarded as the microbiota-gut-brain axis (MGBA). Anxiety (Guo et al., 2019), depression (Liu et al., 2020), Alzheimer’s disease (Sun et al., 2021), Parkinson’s disease (Dong et al., 2020), and autism are among the neurodegenerative disorders linked with gut dysbacteria.

Several studies have shown that surgery/anesthesia stress can lead to gut dysbacteria (Jiang et al., 2019; Liufu et al., 2020; Wen et al., 2020). Other perioperative factors can also affect the microbiota balance. For example, preoperative fasting (Guo et al., 2021), anxiety and insomnia (Neroni et al., 2021), intraoperative trauma stress, antibiotic administration, and blood transfusions (Yracheta et al., 2021), postoperative inflammation (Xia et al., 2021), proton pump inhibitors (PPIs; Haran et al., 2021) and opioid administration (Cruz-Lebron et al., 2021) can all strongly damage the gut microbiota balance; thus, perioperative gut dysbacteria is prevalent. Perioperative neurocognitive disorder (PND), including postoperative delirium (POD) and postoperative cognitive dysfunction (POCD), is a series of severe neurocognitive impairments that can result in long-term cognitive dysfunction, increase the social burden and even mortality (Steinmetz et al., 2009). Importantly, there are no available disease-modifying treatments for PND. The pathophysiology of PND has significant ties to the inflammatory response (IR; Zuo et al., 2021). Gut dysbiosis can cause inflammatory responses in the colon, and the central nervous system. Therefore, several studies focus on the relationship between gut dysbiosis-related IR and PND.

Due to their antimicrobial properties, antibiotics can significantly impact the gut microbiome equilibrium. However, we discovered that cefazolin had an anti-inflammatory impact on C8-B4 cells treated with lipopolysaccharide (Liang et al., 2018). This finding infers that cefazolin, as an antibiotic, may have a double-edge effect on IR, which further affects cognition. Although this double-edged effect was revealed in our previous research, the characteristics of perioperative intestinal dysbacteria and the type of dysbacteriosis that contributed to cognitive disturbance remain unknown. Prospectively, probiotics administration can restore dysbiosis and improve cognitive dysfunction (Eastwood et al., 2021; Roy Sarkar et al., 2021). They could reconstruct the intestinal environment broken by dysbiosis, thus restoring a new balance system.

On the one hand, probiotics could restore the microbiota directly. Conversely, by the production of prebiotics and metabolites, probiotics may have a positive influence on rebuilding. From animal to population studies, researchers demonstrated probiotics’ efficacy in treating PND (Wang P. et al., 2021; Zuo et al., 2021). Our study investigated the double-edged effect of cefazolin on PND, the features of dysbacteria, and whether PND might be treated by restoring intestinal dysbacteriosis following probiotics delivery.

Furthermore, it is crucial to examine the mechanism involved in the probiotics-induced recovery of PND caused by dysbacteria. In recent years, cognition-related research on the pyroptosis pathway has gradually been conducted (Fu et al., 2019; Xu et al., 2019). The pyroptosis pathway is a gasdermin-mediated cell death program that includes the classic NLRP3-related inflammatory pathway mediated by caspase-1. It has been determined that the NLRP3/caspase-1 pathway plays a crucial role in isoflurane-induced cognitive impairment (Wang et al., 2018). Probiotics could ameliorate IgA nephropathy, hyperuricemia, and COVID-19 by improving gut dysbiosis and blunting NLRP3 signaling (Kasti et al., 2021; Tan et al., 2022; Zhao et al., 2022). Uncertainty exists about whether NLRP3 inflammasome activation contributes to perioperative dysbacteria-induced PND and cognitive improvement by probiotics therapy. We speculate that probiotics therapy might effectively reconstruct the balance of the intestinal microbiome, which could somewhat inhibit the NLRP3/caspase-1 pathway, thus suppressing inflammatory responses and ameliorating cognitive impairment.

## Methods

### Animals and experimental group

Six- to 8-week-old Kunming male mice (30–36 g, from Chengdu Dashuo Laboratory Animal Co., LTD) were used. The mice were housed in cages (5 mice/cage) on a 12-h light/dark cycle, with free access to water and food. Mice were put into the fixed condition to adapt for 3 days and then were randomly assigned to each group. For behavior assessment, observers were blinded to the group assignment and the mice treatments.

**Figure 1:**
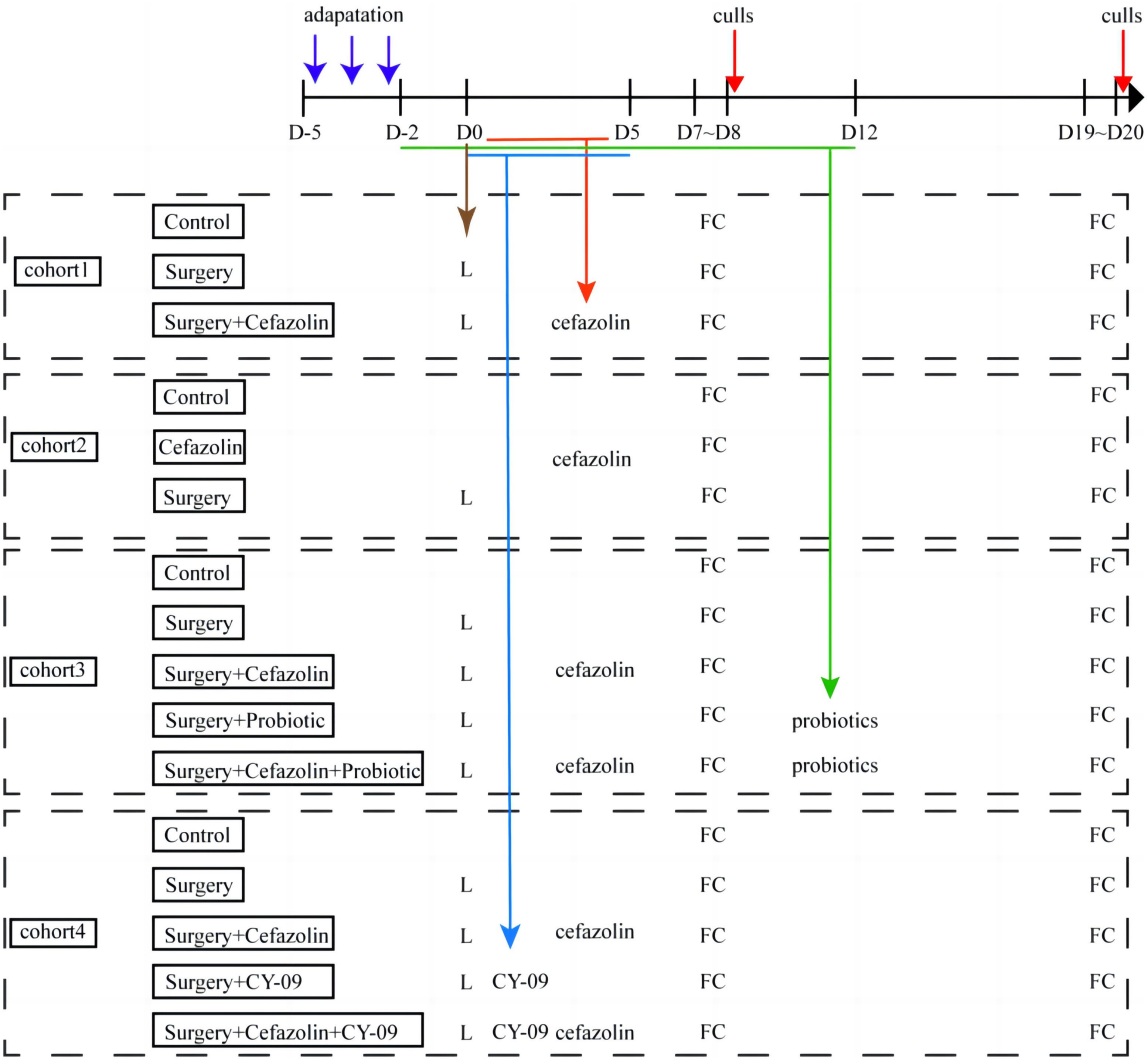
Flow diagram of this study. D, day; L, laparotomy; FC, fear conditioning test (*n* = 20 per group).

Four separate cohorts of mice were used in the experiments (Figure 1). First, the effect of surgery/anesthesia and antibiotics (cefazolin) on postoperative cognitive and dysbacteria was examined. In the first cohort, the mice were randomized by an SPSS-generated random number assignment to 1 of 3 groups (*n* = 20 in each group): control (Grp CTL), surgery (Grp S), surgery combined with cefazolin (Grp SC). These mice were used for FC tests, which started 7 and 19 days after the surgery. After finishing each FC test, mice were humanely killed to obtain the hippocampus and colon for ELISA analyses of Interleukin-1β (IL-1β) and Interleukin-18 (IL-18), and feces for 16S rRNA analyses of the fecal microbiome at 1 or 3 weeks after surgery (*n* = 10 in each group). The second cohort mice (n = 20 in each group) were also assigned to three groups: control (Grp CTL), cefazolin (Grp CEF), and surgery (Grp S) respectively. The same FC tests and sampling methods were employed for the first cohort. The hippocampus and colon for ELISA analyses of IL-1β and IL-18, the hippocampus for PCR analyses of tight junction protein (TJP/ZO-1) mRNA, and feces for 16S rRNA analyses of the fecal microbiome at 1 or 3 weeks after surgery (*n* = 10 in each group) conducted. The possible benefits of probiotics on postoperative cognitive functioning and flora were subsequently examined. The third cohort of mice was randomly assigned into five groups (*n* = 20 in each group): control (Grp CTL); surgery (Grp S); surgery combined with cefazolin (Grp SC); surgery combined with probiotics (Grp SP); and surgery combined with cefazolin and probiotics (Grp SCP). FC tests were used to evaluate the neurocognitive function. 16S rRNA and PCR, ELISA was used to detect the level of IL-1β and IL-18, NLRP3, ZO-1, gut flora following the surgery/anesthesia stress with or without antibiotic or probiotics treatment, respectively at 1 or 3 weeks after surgery (*n* = 10 in each group). Finally, the function of the NLRP3 pathway in perioperative antibiotic-related dysbacteria and cognitive impairment was established. The fourth cohort of mice was also assigned into five groups (*n* = 20 in each group): control (Grp CTL); surgery (Grp S); surgery combined with the NLRP3 inhibitor CY-09 (Grp SI); surgery combined with cefazolin (Grp SC); and surgery combined with cefazolin and CY-09 (Grp SCI). Using the same behavioral tests and sampling methods previously described, the hippocampus and colon were harvested 1- or 3-weeks following surgery for ELISA and PCR analyses of IL-1β and IL-18, NLRP3, caspase-1, and ZO-1.

### Ethics approval and consent to participate

The animal protocol was approved by the Animal Ethics Committee of West China Hospital of Sichuan University (Chengdu, Sichuan, China). All animal experiments followed the Animal Research: reporting of *in vivo* Experiments (ARRIVE) guidelines.

### Exploratory laparotomy model

All mice were deeply anesthetized with 3% sevoflurane (Abbott laboratories) for exploratory laparotomy, with spontaneous respiration retained and a 50% oxygen concentration inhaled. Rectal temperature was monitored and maintained at 37°C with a heated blanket. The abdomen hair was shaved, and the abdominal skin was sterilized with iodophor. Local anesthesia was achieved using ropivacaine (0.25%, 3 mg/kg, AstraZeneca AB) infiltrated into the incision preoperatively. After the abdominal cavity was opened, the right colon, liver, spleen, kidney, and bladder peritoneum were examined using a cotton swab soaked in normal saline (NS). Every mouse was explored using the same set of procedures to ensure a similar degree of stimulation. After the peritoneum and skin were closed successively, each mouse was again subcutaneously injected with ropivacaine (0.25%, 3 mg/kg) for postoperative analgesia. After surgery, the incision was disinfected with iodophor once daily for 5 days to prevent infection. The total anesthesia time was 2 h. For the control and cefazolin-treated mice, neither anesthesia nor surgery was done, which more closely resembles the clinical circumstance in which surgery is performed in conjunction with the anesthesia rather than anesthesia alone.

### Cefazolin administration

Cefazolin (Sinopharm chemical reagent Co., Ltd., 1 g/vial) was dissolved in normal saline to a concentration of 100 mg/mL. As previously described, Cefazolin (300–500 mg/kg) was used to prevent wound infection in mice (Liang et al., 2018). In the study, 10 mg cefazolin in a volume of 0.1 mL was intraperitoneally injected 30 min before surgery and then once a day for 5 days after surgery, while 0.1 mL NS was intraperitoneally injected in the group that did not receive cefazolin.

### Fos and Probiotics administration

Bene-Bac Plus Fos and Probiotics (Bene-Bac Company), a mixture of prebiotics and probiotics for rodents, was administered in our study. Each gram of probiotics powder contained 2 × 10^8^ Colony Forming Units (CFU) of viable bacteria, including *Bifidobacterium bifidum*, *Lactobacillus fermentum*, *L acidophilus*, *L. casei*, *Enterococcus faecium*, *L. plantarum*, and *Pediococcus acidilactici.* Powdered probiotics were dissolved in NS at a concentration of 1 g/ml, with full dissolution requiring water bath kettles maintained at 37°C. Each mouse received an intragastric dose of 1 × 10^8^ CFU in 0.5 mL of probiotic suspension. Solutions were prepared immediately before use. Fos and Probiotics were administered daily for 15 days, beginning 2 days before surgery and concluding 12 days after surgery.

### CY-09 preparation

CY-09 is a selective and direct NLRP3 inhibitor that directly attaches to the ATP-binding motifs of the NLRP3 NACHT domain and thus blocks NLRP3 ATPase activity, inhibiting the assembly and NLRP3 inflammasomes activation. CY-09 (Medchem Express, HY-103666) prepared a 1 ml working solution. A 100 μL clarified DMSO (25.0 mg/mL) reserve solution was mixed with 400 μL PEG300, 50 μL Tween-80, and 450 μL NS was added. A solution ≥ 2.5 mg/mL (5.90 mM, saturation unknown) was obtained with this scheme. Intraperitoneal injections were given at 2.5 mg/kg once daily, 2 days before and 12 days after surgery for 15 days.

### Fear-conditioning tests

FC tests were undertaken 7 days following surgery to investigate the fear memory of mice about context and cue tone. A transparent plastic chamber wiped with 70% alcohol was positioned in a darkened room to accommodate mice encountering three tone-foot shock pairings at 1-min intervals (tone, 2,000 Hz/85 dB/3 s; foot shock, 0.7 mA/1 s). Mice were returned to the same chamber for 7 min without tone and shock 24 h after training. The freezing behavior was recorded during the entire period. After 2 h, mice were placed in another chamber and wiped with 1% acetic acid in a relatively light room with a different context. The tone stimulus was applied for three cycles (3 s per cycle, 2 min inter-cycle interval, total time 7 min) after 2 min without any stimuli delivered to the cage.

### Harvesting of hippocampus and colon

Under sevoflurane anesthesia, mice were killed and perfused with ice-cold PBS to flush blood from the brain vasculature. The bilateral hippocampus and descending colon were removed for q-PCR and ELISA testing. The feces were harvested for 16S rRNA assessment. All dissection procedures were performed on ice, and all samples were stored at −80°C until required for subsequent analysis.

### Fecal sampling and 16S rRNA analyses

Microbial DNA was extracted using the HiPure Stool DNA Kits (Magen, Guangzhou, China) according to the manufacturer’s protocols. The 16S rRNA V3-V4 region of the ribosomal RNA gene was amplified by PCR (95°C for 5 min, followed by 30 cycles at 95°C, 60°C, and 72°C for 1 min each and a final extension at 72°C for 7 min)using primers (341F: CCTACGGGNGGCWGCAG; 806R: GGACTACHVG GGTATCTAAT; Guo et al., 2017). PCR reactions were performed in triplicate 50 μL mixture containing 10 μL of 5 × Q5@ Reaction Buffer, 10 μL of 5 × Q5@ High GC Enhancer, 1.5 μL of 2.5 mM dNTPs, 1.5 μL of each primer (10 μM), 0.2 μL of Q5@ High-Fidelity DNA Polymerase, and 50 ng of template DNA. Related PCR reagents were from New England Biolabs, United States.

Raw data containing adapters or low-quality reads would impact the following assembly and analysis. Thus, to get high-quality clean reads, raw reads were further filtered according to the following rules using FASTP (Chen et al., 2018; version 0.18.0): (1) Removing reads containing more than 10% of unknown nucleotides (N); (2) Removing reads containing less than 50% of bases with quality (Q-value) ˃ 20. Paired-end clean reads were merged as raw tags using FLSAH (Magoc and Salzberg, 2011; version 1.2.11) with a minimum overlap of 10 bp and mismatch error rates of 2%. The representative OTU sequences were classified into organisms by a naive Bayesian model using an RDP classifier (Wang et al., 2007; version 2.2) based on the SILVA database (Pruesse et al., 2007; version 132), with the confidence threshold value of 0.8.

A comparative quantitative evaluation of gut microbiota was conducted using agarose gel electrophoresis. Bacterial diversity analysis depended on 16S rRNA library sequencing, performed on the Illumina HiSeq2500 by Gene Denovo Biotechnology Co., Ltd. (Guangzhou, China). Bioinformatic analysis was conducted using Omicsmart, a real-time interactive online platform for data analysis^1^ (Peng et al., 2022; Wu et al., 2022). If researchers have any requirements for the raw reads, you can also contact us by author’s email (liangpengwch@scu.edu.cn).

The KEGG pathway analysis of the OTUs was inferred using Tax4Fun (Asshauer et al., 2015; version 1.0) or PICRUSt (Langille et al., 2013; version 2.1.4). Microbiome phenotypes of bacteria were classified using BugBase (Ward et al., 2017). Analysis of function difference between groups was calculated by Welch’s t-test, Wilcoxon rank test, Kruskal Wallis H test, and Tukey’s HSD test in the R project Vegan package (Oksanen et al., 2011; version 2.5.3).

### ELISA of IL-1β and IL-18 in the hippocampus and colon

IL-1β and IL-18 in the hippocampus and colon were detected using ELISA kits (ExCell Bio., China). Tissues (hippocampus or colon) were homogenized on ice in a PBS buffer. After being centrifuged for 20 min (13,000 g, 4°C), the supernatant was collected for ELISA detection according to the manufacturer’s instructions. Each sample’s IL-1β and IL-18 concentrations were adjusted based on protein content. The total protein concentration was measured using BCA kits (Beyongtime., China).

### PCR of ZO-1, NLRP3, caspase-1, IL-1β and IL-18 in hippocampus and colon

Tissues from the colon and hippocampus were extracted. According to the manufacturer’s instructions, RNA was isolated from total RNA using an RNA isolation kit (Qiagen, Inc., Carlsbad, CA, United States). A total of 1,000 ng of RNA was used as the RNA template for reverse transcription using GoScript™ Reverse Transcription Mix, Oligo (dT) with the following procedure: 25°C for 5 min, 42°C for 60 min, and 70°C for 15 min. The primers for amplification are listed in Table 1. The substrate for amplification was the GoTaq® qPCR Master Mix (Promega, A6002, USA). The amplification procedure was followed: 95°C for 5 min, 40 cycles at 95°C for 15 s, and 60°C for 1 min. The fluorescent data were collected on every cycle and analyzed.

**Table 1:** Pearson’s correlation analysis between G^+^/G^−^ ratio and targeted indicators.

Indicator	Located tissue	Pearson’s correlation	R^2^	Linear formula	*P*-value
IL-1β	In colon	0.86	0.739	y = 1.682X + 0.516	0.001^*^
	In hippocampus	0.852	0.726	y = 0.913x + 0.599	0.002^*^
IL-18	In colon	0.817	0.667	y = 3.001x + 0.233	0.004^*^
	In hippocampus	0.746	0.557	y = 0.668x + 0.74	0.013^*^
NLRP3	In colon	0.847	0.718	y = 1.557x + 0.464	0.002^*^
	In hippocampus	0.88	0.775	y = 0.955x + 0.677	0.001^*^
ZO-1	In colon	0.828	0.685	y = −0.368X + 1.147	0.003^*^
	In hippocampus	0.752	0.566	y = −0.176X + 1.026	0.012^*^

* *p* < 0.05.

### Statistical analysis

GraphPad Prism Version 8.2.1 (441) was used for graphics, and SPSS ver. 21.0 for statistical analyses. Continuous variables are presented as means ± SEM (*n* ≥ 6). Classified or hierarchical variables are given as the frequency (percentage). When a normal distribution and homogeneity of variance were satisfied, one-way analysis of variance (ANOVA) followed by Tukey’s multiple comparison tests was used to compare data between groups. The Brown-Forsythe test was used to determine whether the variance was homogeneous. When the homogeneity of variance was not satisfied, the Welch test was employed. The Kolmogorov–Smirnov test was used to determine whether a normal distribution was present. When the distribution was abnormal, a Kruskal-Wallis test followed by Dunn’s multiple comparison tests was conducted to analyze the data between groups. The Kruskal-Wallis test, followed by the Wilcoxon test, was used to compare multi-group grade data and analyze intestinal flora diversity. *p-*value < 0.05 was statistically significant.

## Results

### Cefazolin alleviated fear memory deficits induced by surgery/anesthesia stress 1 week after surgery but aggravated it at 3 weeks

**Figure 2:**
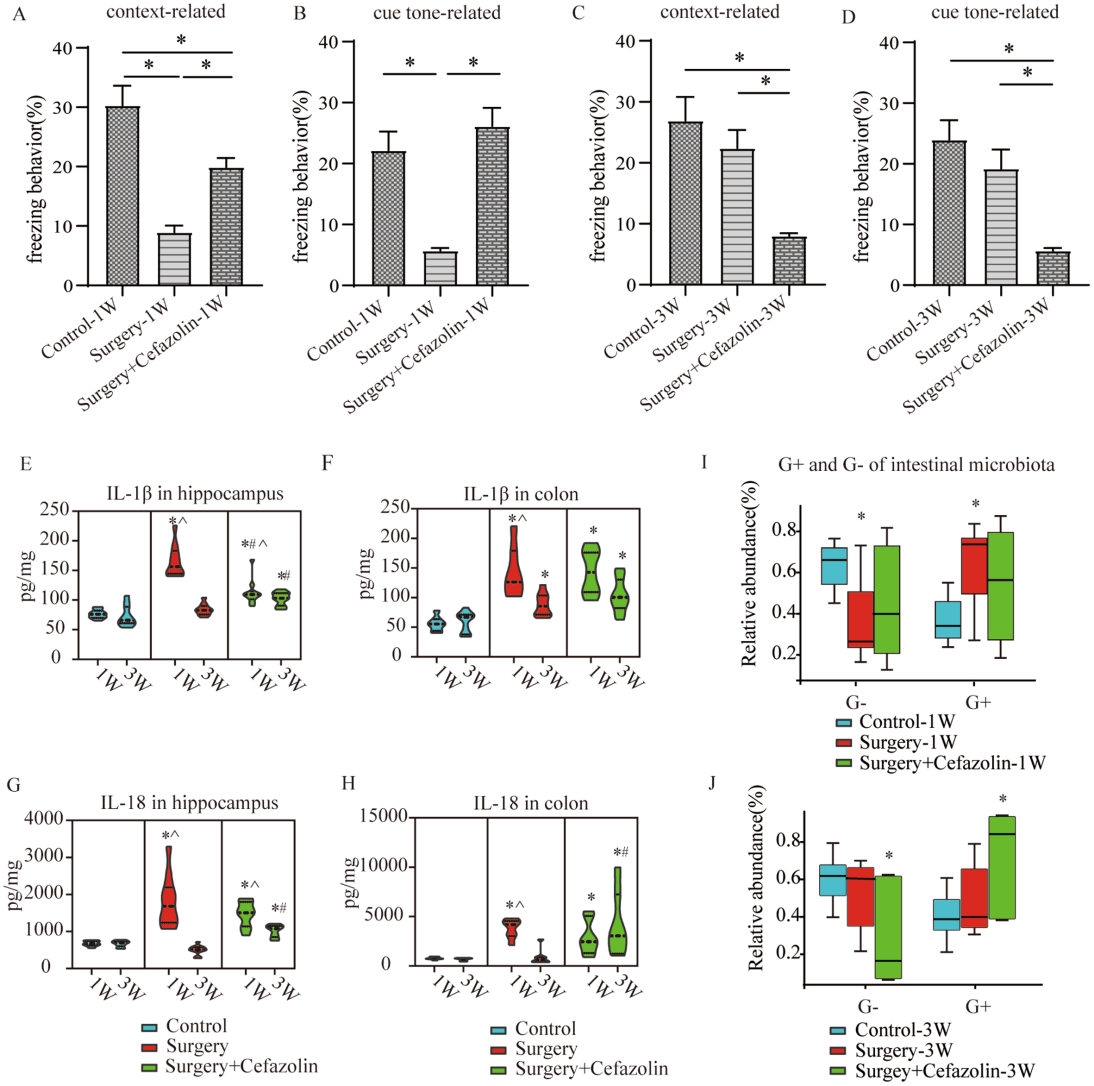
Surgery/anesthesia stress-induced and perioperative cefazolin-induced gut dysbacteria, inflammation, and cognitive dysfunction, respectively. **(A,B)** Surgery/anesthesia stress decreased context-related (q = 9.737, *p* < 0.0001) and cue tone-related (q = 6.635, *p* = 0.0002) freezing behavior at 1 week, while cefazolin could improve context-related (q = 4.996, *p* = 0.0041) and cue tone-related (q = 8.229, *p* < 0.0001) freezing behavior (*n* = 10, ANOVA with Tukey’s multiple comparisons test). **(C,D)** At 3 weeks after surgery, surgery/anesthesia stress-induced freezing behavior recovered, while cefazolin decreased context-related (vs. control, q = 6.671, *p* = 0.0002; vs. surgery, q = 5.078, *p* = 0.0036) and cue tone-related (vs. control, q = 7.029, *p* < 0.0001; vs. surgery, q = 5.193, *p* = 0.0029) freezing behavior (*n* = 10, ANOVA with Tukey’s multiple comparisons test). **(E,F)** ELISA: surgery/anesthesia stress increased the level of IL-1β in the hippocampus (q = 13.66, *p* < 0.0001) and colon (q = 6.832, *p* = 0.0004) at 1 week, respectively; at 3 weeks, only IL-1β in the colon (q = 4.177, *p* = 0.0171) was elevated by surgery/anesthesia stress; cefazolin could alleviate increasing IL-1β in the hippocampus (q = 8.005, *p* < 0.0001) at 1 week, but at 3 weeks, could enhance IL-1β in the hippocampus (q = 6.925, *p* = 0.0001) and colon (q = 6.437, *p* = 0.0003), respectively (n = 10, ANOVA with Tukey’s multiple comparisons test). **(G,H)** ELISA: surgery/anesthesia stress increased the level of IL-18 in the hippocampus (q = 7.902, *p* < 0.0001) and colon (q = 6.803, *p* = 0.0004) at 1 week, respectively; at 3 weeks, cefazolin could enhance IL-18 in the hippocampus (q = 8.352, *p* < 0.0001) and colon (q = 4.697, *p* = 0.0101), respectively (*n* = 10, ANOVA with Tukey’s multiple comparisons test). **(I,J)** Surgery/anesthesia stress increased the relative abundance of gram-positive bacteria (q = 0.0638, *p* = 0.0142) and decreased the relative abundance of gram-negative bacteria (q = 0.0638, *p* = 0.0142) of intestinal microbiota at 1 week. In contrast, cefazolin increased the relative abundance of gram-positive bacteria (q = 0.0733, *p* = 0.0244). It decreased the relative abundance of gram-negative bacteria (q = 0.0733, *p* = 0.0244) of intestinal microbiota at 3 weeks after surgery (*n* = 9, KW rank sum test with Wilcoxon rank sum test). G^+^: gram-positive bacteria; G^−^: gram-negative bacteria. ^*^Compared with control, *p* < 0.05; ^#^compared with surgery, *p* < 0.05; ^^^compared with 3 weeks, *p* < 0.05.

The time of freezing behavior for mice in the FC test decreased in context-related and tone-related FC 1 week after surgery (Figures 2A,B). Surprisingly, cefazolin greatly enhanced these surgical/anesthesia-induced decreases in freezing behavior. Three weeks after surgery, the surgery group improved context- and tone-related freezing behavior. However, cefazolin aggravated the declination of freezing behavior and delayed the recovery of cognitive functions (Figures 2C,D). These data revealed that cefazolin caused paradoxical effects on postoperative cognitive impairment. Why do paradoxical effects emerge?

### The double-edge effect of cefazolin on PND was correlated with inflammation in the hippocampus and the gram-positive bacteria/gram-negative bacteria ratio in intestinal flora

One week following surgery, perioperative cefazolin treatment enhanced context-related freezing behavior. However, it oppositely inhibited the recovery of freezing behavior 3 weeks after surgery. Similarly, cefazolin decreased IL-1β and IL-18 levels in the hippocampus 1 week after surgery and increased levels in the colon and hippocampus 3 weeks after surgery (Figures 2E–H). These results suggested that the levels of proinflammatory cytokines, particularly in the hippocampus, might be associated with cognitive changes.

**Figure 3:**
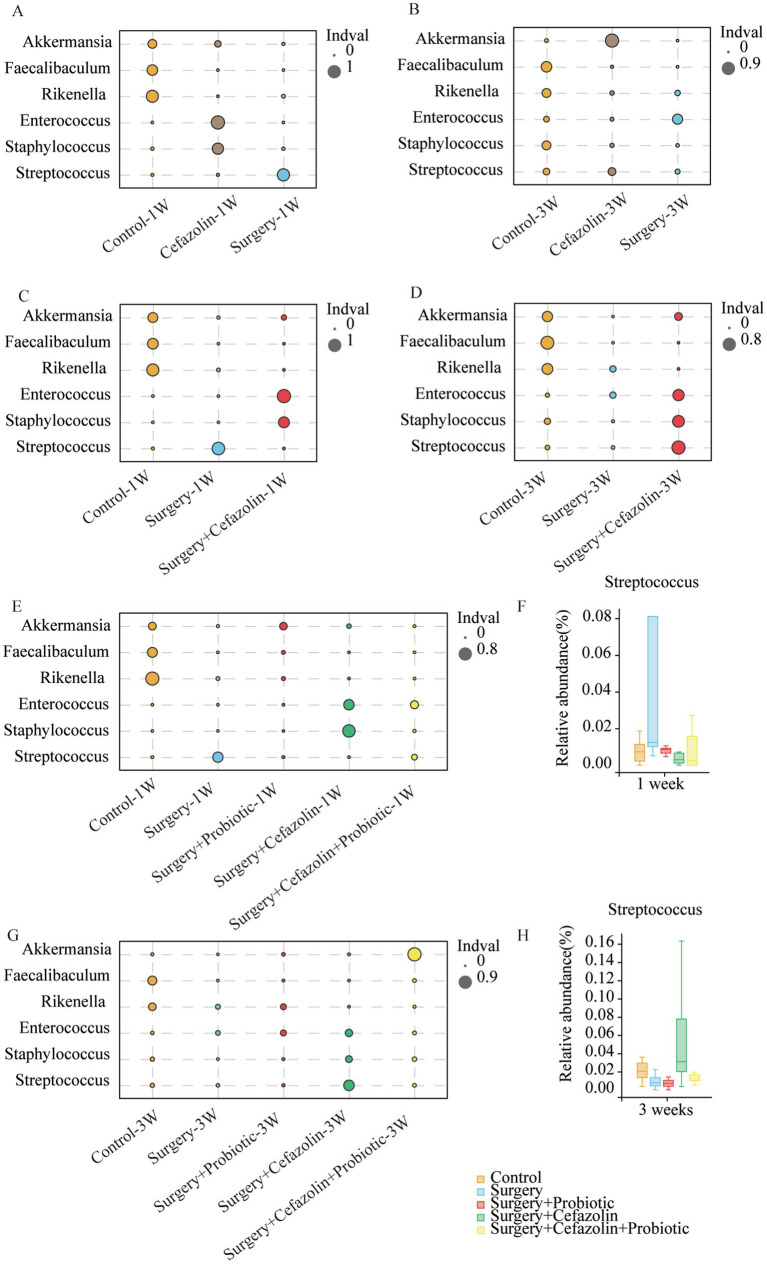
The probiotics administration for restoring surgery/anesthesia stress-induced and perioperative cefazolin-induced gut dysbacteria, respectively. **(A,B)** The differential species analysis in the genus level of Grp CTL, Grp CEF, Grp S at 1 and 3 weeks after surgery; **(C,D)** The differential species analysis in the genus level of Grp CTL, Grp S, Grp SC at 1 week and 3 weeks after surgery; **(E,G)** The differential species analysis in the genus level of Grp CTL, Grp S, Grp SP, Grp SC, Grp SCP at 1 and 3 weeks after surgery; **(F,H)** Surgery/anesthesia stress increased the relative abundance of Streptococcus at 1 week (q = 0.7693, *p* = 0.0399) while probiotics could decrease it (q = 0.5489, *p* = 0.0314); cefazolin could increase the relative abundance of Streptococcus at 3 weeks after surgery (q = 0.3125, *p* = 0.0314) compared with surgery group (*n* = 9, Wilcoxon rank sum test). Grp, group; CTL, control; S, surgery; SC, surgery combined with cefazolin; SP, surgery combined with probiotics; SCP, surgery combined with cefazolin and probiotics.

Differential species analysis showed taxa with significant group differences. At the genus level, surgery enhanced the abnormal proliferation of *Streptococcus* at 1 week, while perioperative cefazolin suppressed this improvement (Figure 3C). At 3 weeks, however, the surgery/anesthesia-induced *Streptococcus* growth progressively returned; however, cefazolin enhanced the *Streptococcus* increase (Figure 3D). *Streptococcus* is one of Gram-positive bacterium; hence we also found some changes in the G^+^/G^−^ ratio (Figures 2I,J). The increasing trends of the G^+^/G^−^ ratio were in line with the declination of cognition.

**Figure 4:**
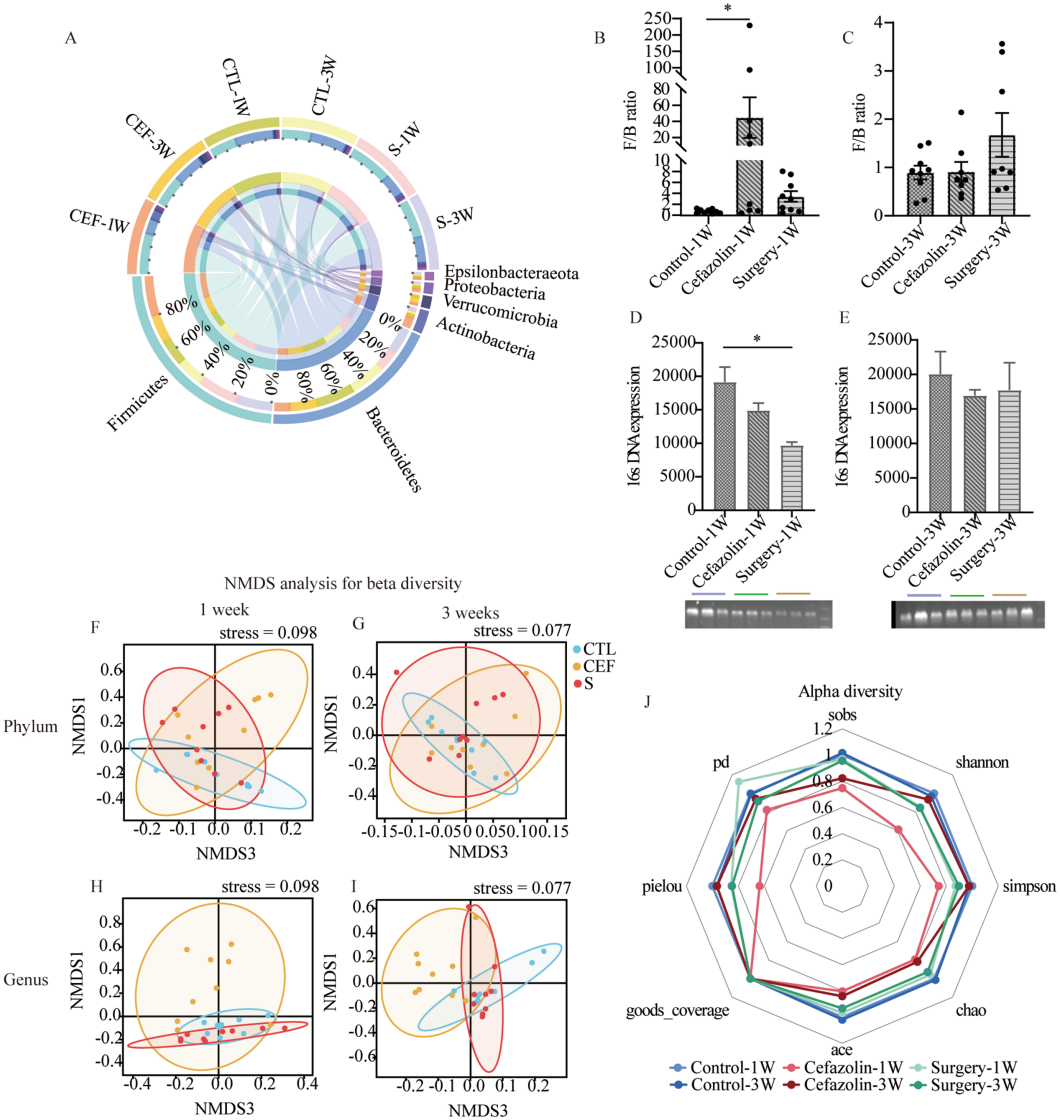
Cefazolin-induced and surgery/anesthesia stress-induced gut dysbacteria. **(A)** The circus graph of the composition of the intestinal microbiome at the level of a phylum of Grp CTL, Grp CEF, and Grp S at 1 and 3 weeks; **(B)** The F/B ratio of the intestinal microbiome in the level of a phylum of Grp CTL, Grp CEF, Grp S at 1 week; **(C)** The F/B ratio of the intestinal microbiome in the level of a phylum of Grp CTL, Grp CEF, Grp S at 3 weeks; **(D)** The 16S rRNA expression of the gut microbiome of Grp CTL, Grp CEF, Grp S at 1 week; **(E)** The 16S rRNA expression of the gut microbiome of Grp CTL, Grp CEF, Grp S at 3 weeks. **(F)** Comparison between intra-group and inter-group Bray-Curtis distance of gut microbiome in the phylum level at 1 week of Grp CTL, Grp CEF, Grp S; **(G)** Comparison between intra-group and inter-group Bray-Curtis distance of gut microbiome in the phylum level at 3 weeks of Grp CTL, Grp CEF, Grp S; **(H)** Comparison between intra-group and inter-group Bray-Curtis distance of gut microbiome in the genus level at 1 week of Grp CTL, Grp CEF, Grp S; **(I)** Comparison between intra-group and inter-group Bray-Curtis distance of gut microbiome in the genus level at 3 weeks of Grp CTL, Grp CEF, Grp S. **(J)** The radar graph of alpha diversity of gut microbiome of Grp CTL, Grp CEF, Grp S at 1 and 3 weeks. Grp, group; CTL, control; CEF, cefazolin; S, surgery; F/B, Firmicutes/Bacteroidetes; NMDS, non-metric multi-dimensional scaling. ^*^Compared with Grp CTL, *p* < 0.05.

Simultaneously, it was discovered that surgery/anesthesia stress and cefazolin alone can disrupt the equilibrium of gut flora, although each has its unique properties. Cefazolin decreased the diversity of the microbiota, while surgery/ anesthesia stress reduced the number of bacteria (Figure 4).

Therefore, the disruption and restoration of the bacterial population may be associated with mice’s cognitive deficiencies and recovery.

### IL-1β is maintained at a high level in the colon for at least 3 weeks after surgery

**Figure 5:**
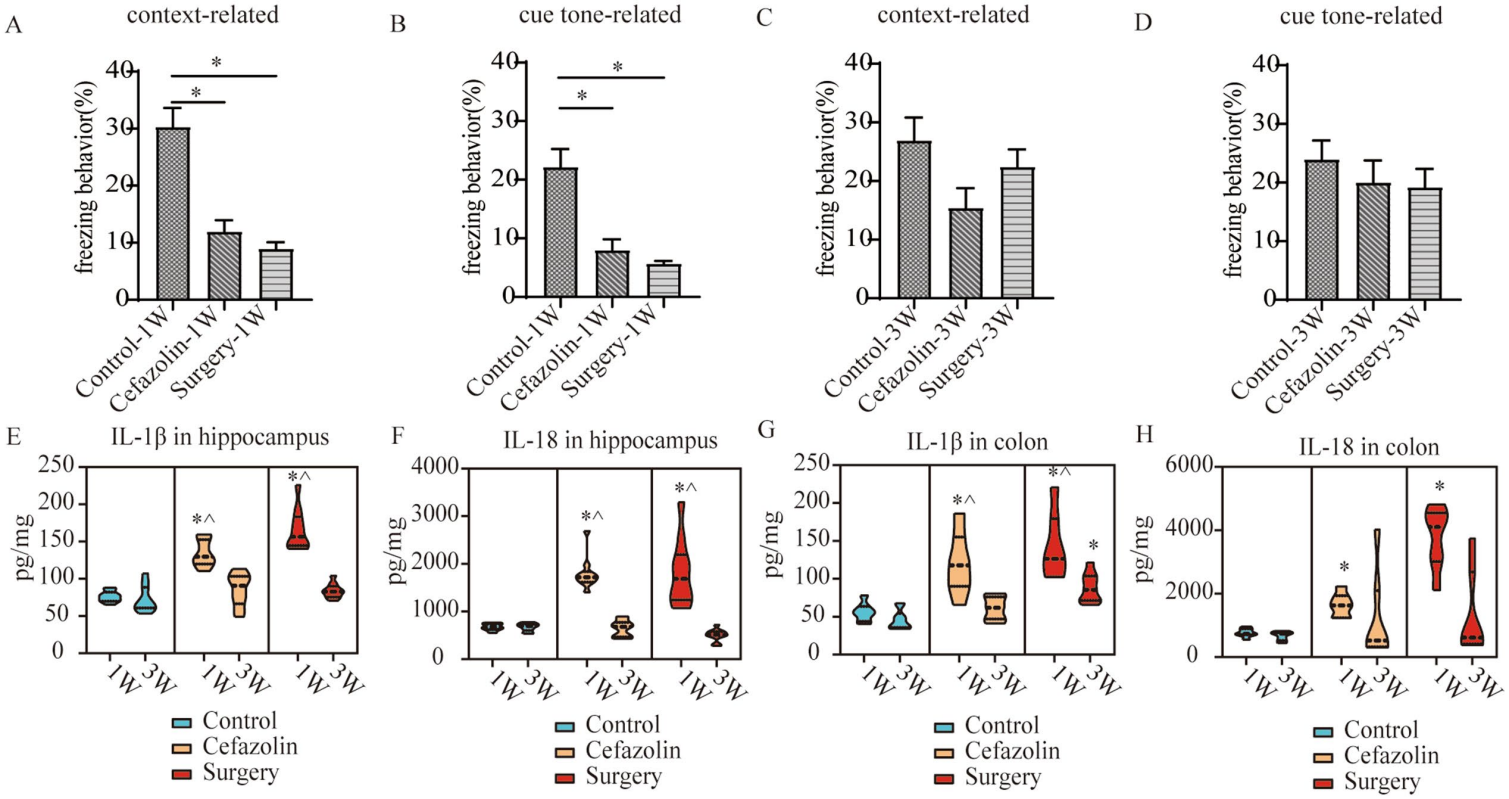
Cefazolin-induced and surgery/anesthesia stress-induced inflammation and cognitive dysfunction, respectively. **(A)** Context-related freezing behavior at 1 week of Grp CTL, Grp CEF, Grp S; **(B)** Cue tone-related freezing behavior at 1 week of Grp CTL, Grp CEF, Grp S; **(C)** Context-related freezing behavior at 3 weeks of Grp CTL, Grp CEF, Grp S; **(D)** Cue tone-related freezing behavior at 3 weeks of Grp CTL, Grp CEF, Grp S; **(E)** ELISA for the level of IL-1β in the hippocampus at 1 and 3 weeks of Grp CTL, Grp CEF, Grp S; **(F)** ELISA for the level of IL-18 in the hippocampus at 1 and 3 weeks of Grp CTL, Grp CEF, Grp S; **(G)** ELISA for the level of IL-1β in the colon at 1 and 3 weeks of Grp CTL, Grp CEF, Grp S; **(H)** ELISA for the level of IL-18 in the colon at 1 and 3 weeks of Grp CTL, Grp CEF, Grp S. Grp, group; CTL, control; CEF, cefazolin; S, surgery. ^*^Compared with 3 weeks, *p* < 0.05.

IL-1β and IL-18 levels in colonic tissue were increased 1 week after surgery or cefazolin administration. Simultaneously, IL-1β and IL-18 levels in the hippocampus were increased. With the reconstruction of dysbiosis, IL-1β and IL-18 levels in the colon and hippocampus were gradually reduced to normal 3 weeks, except for IL-1β in the colon after surgery (Figures 5E–H). Notably, the IL-1β level in the colon 3 weeks after surgery was higher than Grp CTL (Figure 5G), indicating that a high level of IL-1β would continue in the postoperative colon for at least 3 weeks.

### Perioperative probiotics administration adjusted the ratio of G^+^/G^−^ by suppressing the increasing relative abundance of *Streptococcus* and thus improved cognitive impairment

**Figure 6:**
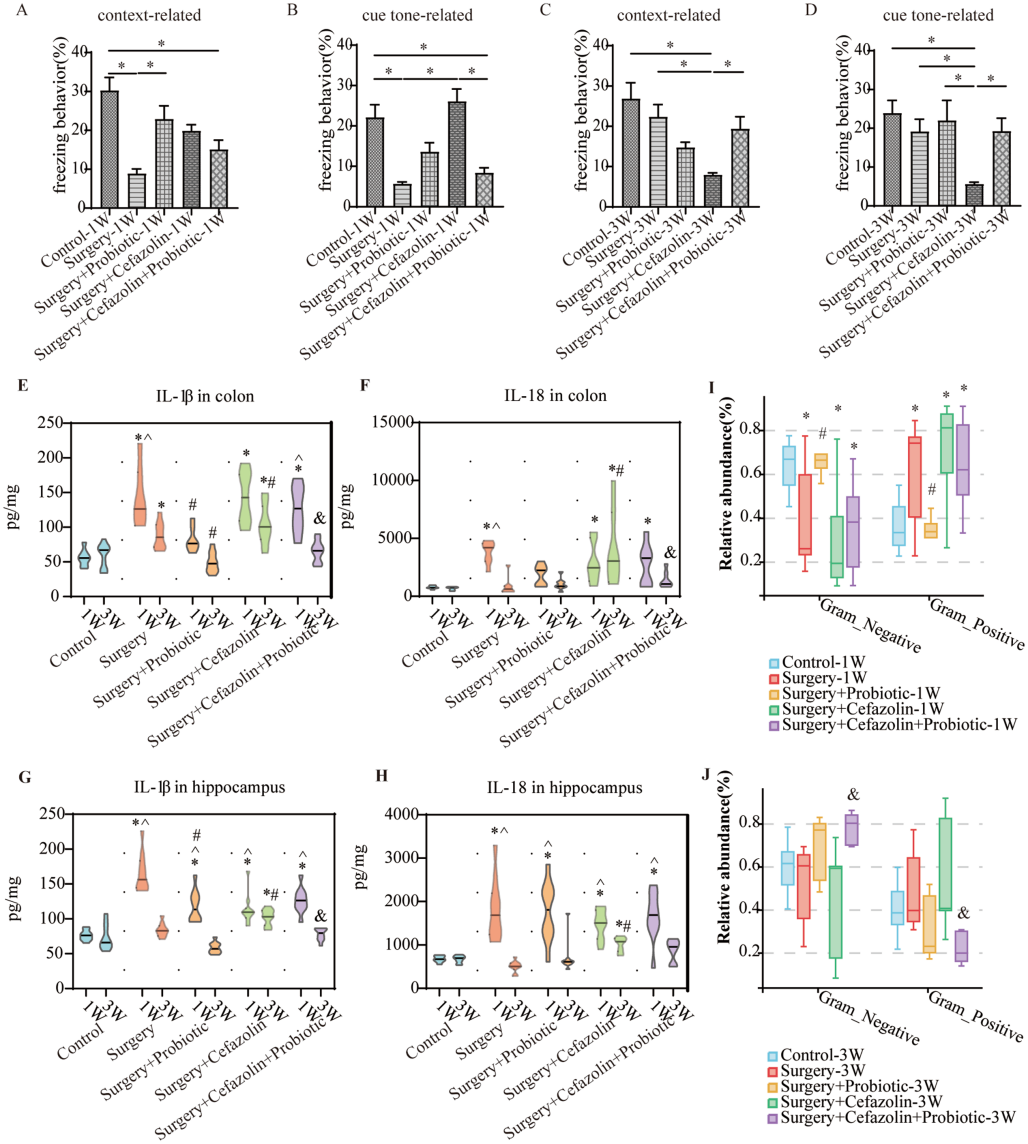
The probiotics administration for surgery/anesthesia stress-induced and perioperative cefazolin-induced gut dysbacteria, and cognitive dysfunction, respectively. **(A,B)** Probiotics improved context-related (q = 5.58, *p* = 0.0024) but not cue tone-related (q = 3.597, *p* = 0.099) freezing behavior decreased by surgery/anesthesia stress at 1 week (*n* = 10, ANOVA with Tukey’s multiple comparisons test). **(C,D)** Surgery + cefazolin group showed a decreasing context-related (q = 6.984, *p* = 0.0001) and cue tone-related (q = 5.406, *p* = 0.0035) freezing behavior compared with the control group at 3 weeks; probiotics could improve these context-related (q = 4.224, *p* = 0.035) and cue tone-related (q = 4.024, *p* = 0.0495) freezing behavior (*n* = 10, ANOVA with Tukey’s multiple comparisons test). **(E,F)** ELISA: probiotics inhibited the increasing IL-1β (q = 5.183, *p* = 0.0078) in the colon at 1 week caused by surgery; probiotics inhibited the increasing IL-1β (q = 6.238, *p* = 0.0006) and IL-18 (q = 4.601, *p* = 0.0219) in the colon at 3 weeks after surgery compared with surgery + cefazolin group (*n* = 10, ANOVA with Tukey’s multiple comparisons test). **(G,H)** ELISA: probiotics inhibited the increasing IL-1β (q = 5.183, *p* = 0.0078) in the hippocampus at 1 week caused by surgery; probiotics inhibited the increasing IL-1β (q = 6.653, *p* = 0.0002) in the hippocampus at 3 weeks after surgery compared with surgery + cefazolin group (*n* = 10, ANOVA with Tukey’s multiple comparisons test). **(I,J)** Probiotics could reverse the increasing relative abundance of gram-positive bacteria (q = 0.0083, *p* = 0.0028) and decreasing gram-negative bacteria (q = 0.0083, *p* = 0.0028) caused by surgery at 1 week, but not surgery + cefazolin; probiotics could reverse the increasing relative abundance of gram-positive bacteria (q = 0.0055, *p* = 0.0018) and decreasing gram-negative bacteria (q = 0.0055, *p* = 0.0018) caused by surgery + cefazolin at 3 weeks (*n* = 9, KW rank sum test with Wilcoxon rank sum test). Grp, group; CTL, control; S, surgery; SC, surgery combined with cefazolin; SP, surgery combined with probiotics; SCP, surgery combined with cefazolin and probiotics; G^+^, gram-positive bacteria; G^−^, gram-negative bacteria. ^*^Compared with Grp CTL, *p* < 0.05; ^#^compared with Grp S, *p* < 0.05; ^&^compared with Grp SC, *p* < 0.05; ^^^compared with 3 weeks, *p* < 0.05.

One week after surgery, perioperative administration of probiotics may mitigate context-related cognitive abnormalities produced by surgery/anesthesia stress (Figure 6A). Moreover, differential species analysis revealed that the relative abundance of *Streptococcus* gradually recovered after administration of probiotics (Figures 3E,F), which maintained a normal standard G^+^/G^−^ ratio (Figure 6I). It was evident that probiotics had the same effect as cefazolin on cognitive improvement 1 week following surgery. The significant association between cognition, *Streptococcus*, and the G^+^/G^−^ ratio, was confirmed again.

### Perioperative antibiotic therapy had a strong impact on the intestinal flora reconstruction produced by probiotics

**Figure 7:**
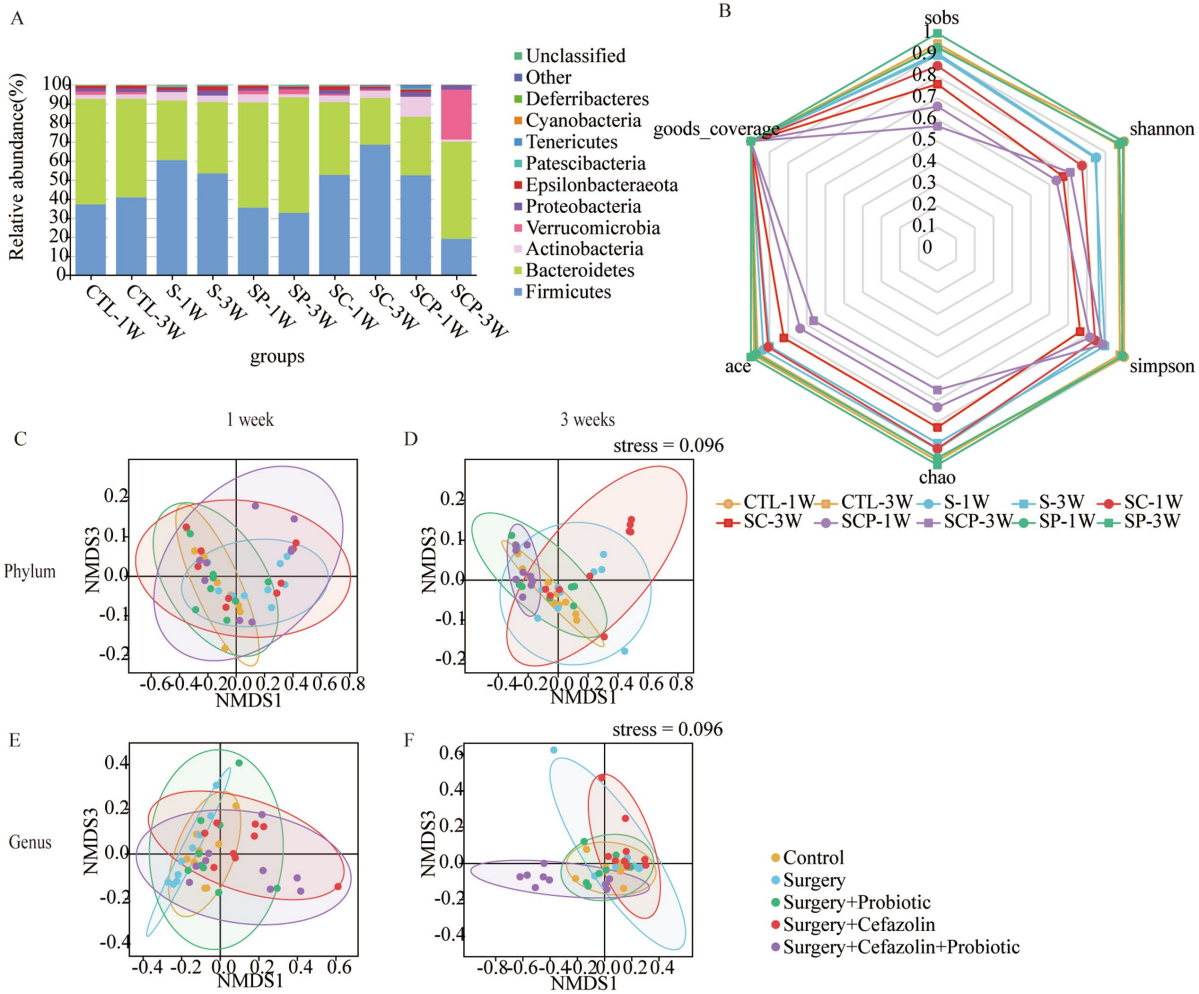
The probiotics administration for restoring surgery/anesthesia stress-induced and perioperative cefazolin-induced gut dysbacteria, respectively. **(A)** The intestinal microbiome composition in the phylum level at 1 and 3 weeks of Grp CTL, Grp S, Grp SP, Grp SC, and Grp SCP. **(B)** The radar graph of alpha diversity of gut microbiome of Grp CTL, Grp S, Grp SP, Grp SC, and Grp SCP at 1 and 3 weeks. **(C–F)** NMDS analysis for beta diversity of gut microbiome: comparison between intra-group and inter-group Bray-Curtis distance of gut microbiome in the phylum level or genus level at 1 or 3 weeks of Grp CTL, Grp S, Grp SP, Grp SC, Grp SCP. Grp, group; CTL, control; S, surgery; SC, surgery combined with cefazolin; SP, surgery combined with probiotics; SCP, surgery combined with cefazolin and probiotics; NMDS, non-metric multi-dimensional scaling.

The administration of probiotics did not come into play during antibiotic therapy. Regarding bacterial composition, the relative abundance of Firmicutes and Bacteroidetes was comparable in the Grp SC and Grp SCP 1 week after surgery. However, sustainable probiotics administration might alter the abundance of Firmicutes and Bacteroidetes 3 weeks after surgery (Figure 7A). Meanwhile, Grp SCP showed a lower alpha diversity of sobs, Chao1, ACE, Shannon, and Simpson, even though a relatively low alpha diversity was maintained as Grp SC 3 weeks after surgery (Figure 7B). Surgery combined with cefazolin with or without probiotics exhibited no differential beta diversity at the phylum and genus levels (Figures 7C,E), but a significant difference 3 weeks after surgery (Figures 7D,F). These results revealed that the anti-microbiome action of cefazolin injection significantly impacted the colonization of probiotics. Alternately, continuing probiotics treatment for 7 days following cefazolin suspension would effectively correct the microbiome’s composition but not its diversity.

Probiotics did not ameliorate the declination of freezing behavior in the surgery + cefazolin group 1 week after surgery (Figures 6A,B). However, at postoperative 3 weeks, probiotics significantly attenuated the cognitive deficits related to surgery when combined with cefazolin (Figures 6C,D). Probiotics can reduce the increasing abundance of *Streptococcus* induced by surgery/anesthesia stress (Figures 3E,F). Furthermore, probiotics reduced the proliferation of *Streptococcus* induced by surgery paired with cefazolin 3 weeks after surgery (Figures 3G,H). Moreover, a high abundance of Akkermansia appeared as a specific kind of bacteria (Figure 3H). *Akkermansia* is an emerging G^−^ which has been studied extensively. The rectification of the G^+^/G^−^ ratio in Grp SCP, 3 weeks after surgery, was facilitated by the decreasing *Streptococcus* and increasing *Akkermansia* populations (Figures 6I,J). Considering these data, it is evident that probiotics can ameliorate the frozen behavior deficit caused by cefazolin 3 weeks following surgery.

### The G^+^/G^−^ ratio change was related to NLRP3-related inflammation and the permeability of the blood–brain barrier and the enteric epithelial barrier

**Figure 8:**
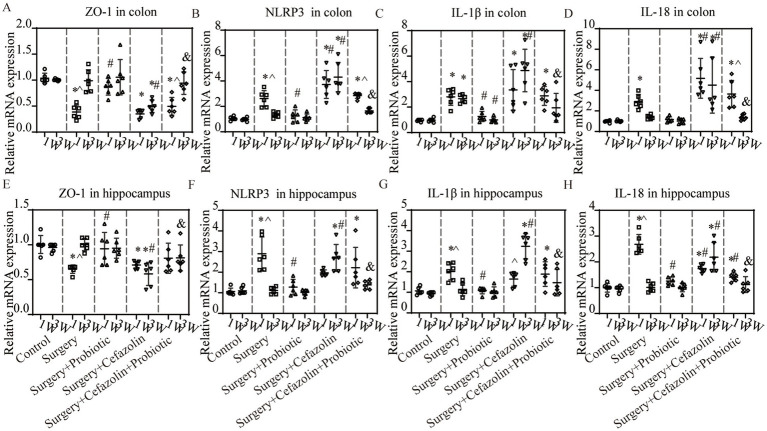
The probiotics administration for surgery/anesthesia stress-induced and perioperative cefazolin-induced inflammatory response and barrier permeability, respectively. **(A,E)** PCR: Surgery/anesthesia stress decreased the level of ZO-1 in the colon (q = 11.99, *p* < 0.0001) and hippocampus (q = 5.474, *p* = 0.0057) at 1 week, while probiotics could recover these decreases (ZO-1 in the hippocampus, q = 4.521, *p* = 0.0281; ZO-1 in the colon, q = 8.955, *p* < 0.0001); at 3 weeks after surgery, surgery + cefazolin group showed a decreasing level of ZO-1 in the colon (q = 5.874, *p* = 0.0028) and hippocampus (q = 7.139, *p* = 0.0003), while probiotics could increase these decreases of ZO-1 in the colon (q = 5.156, *p* = 0.0098) and hippocampus (q = 7.391, *p* = 0.0002; *n* = 6, ANOVA with Tukey’s multiple comparisons test). **(B–D)** PCR: Surgery/anesthesia stress increased the level of NLRP3 (q = 6.262, *p* = 0.0014), IL-1β (q = 5.299, *p* = 0.0077), IL-18 (q = 4.343, *p* = 0.0372) in the colon at 1 week, while probiotics could recover some decreases (NLRP3, q = 5.434, *p* = 0.0061; IL-1β, q = 4.389, *p* = 0.0346); at 3 weeks after surgery, surgery + cefazolin group showed an increasing level of NLRP3 (q = 14.83, *p* < 0.0001), IL-1β (q = 10.37, *p* < 0.0001), IL-18 (q = 6.934, *p* = 0.0004) in the colon, while probiotics could decrease these increases (NLRP3, q = 11.87, *p* < 0.0001; IL-1β, q = 7.807, *p* < 0.0001; IL-18, q = 6.224, *p* = 0.0015; *n* = 6, ANOVA with Tukey’s multiple comparisons test). **(E–H)** PCR: Surgery/anesthesia stress increased the level of NLRP3(q = 7.443, *p* = 0.0002), IL-1β (q = 6.187, *p* = 0.0016), IL-18 (q = 18.49, *p* < 0.0001) in the hippocampus at 1 week, while probiotics could recover some decreases (NLRP3, q = 6.557, *p* = 0.0008; IL-1β, q = 6.060, *p* = 0.002; IL-18, q = 15.65, *p* < 0.0001); at 3 weeks after surgery, surgery + cefazolin group showed an increasing level of NLRP3 (q = 11.83, *p* < 0.0001), IL-1β (q = 12.87, *p* < 0.0001), IL-18 (q = 9.592, *p* < 0.0001) in the hippocampus, while probiotics could decrease these increases (NLRP3, q = 9.682, *p* < 0.0001; IL-1β, q = 9.878, *p* < 0.0001; IL-18, q = 8.299, *p* < 0.0001; *n* = 6, ANOVA with Tukey’s multiple comparisons test). ^*^Compared with Grp CTL, *p* < 0.05; ^#^compared with Grp S, *p* < 0.05; ^&^compared with Grp SC, *p* < 0.05; ^^^compared with 3 weeks, *p* < 0.05.

What changes occurred after probiotics regulated the postoperative gut flora? We further observed inflammation of the colon and hippocampus. The NLRP3 inflammasome was activated in the colon and hippocampus 1 week after surgery, while probiotics had a repressive effect on this activation (Figures 8B,F). Simultaneously, the increasing IL-1β and IL-18 levels in the colon and hippocampus caused by surgery/anesthesia stress were ameliorated by probiotics (Figures 6E–H, 8C,D,G,H). Additionally, probiotics significantly inhibited the decrease of ZO-1 expression in the colon and hippocampus (Figures 8A,E). Notably, 3 weeks after surgery, probiotics ameliorated the perioperative cefazolin-induced long-term inflammation of the colon and hippocampus and restored ZO-1 expression (Figures 8A,E). These inflammatory reactions might be closely related to adjusting the G^+^/G^−^ ratio, which we previously mentioned (Table 1).

### PND was improved by inhibiting dysbiosis-related NLRP3 activation

**Figure 9:**
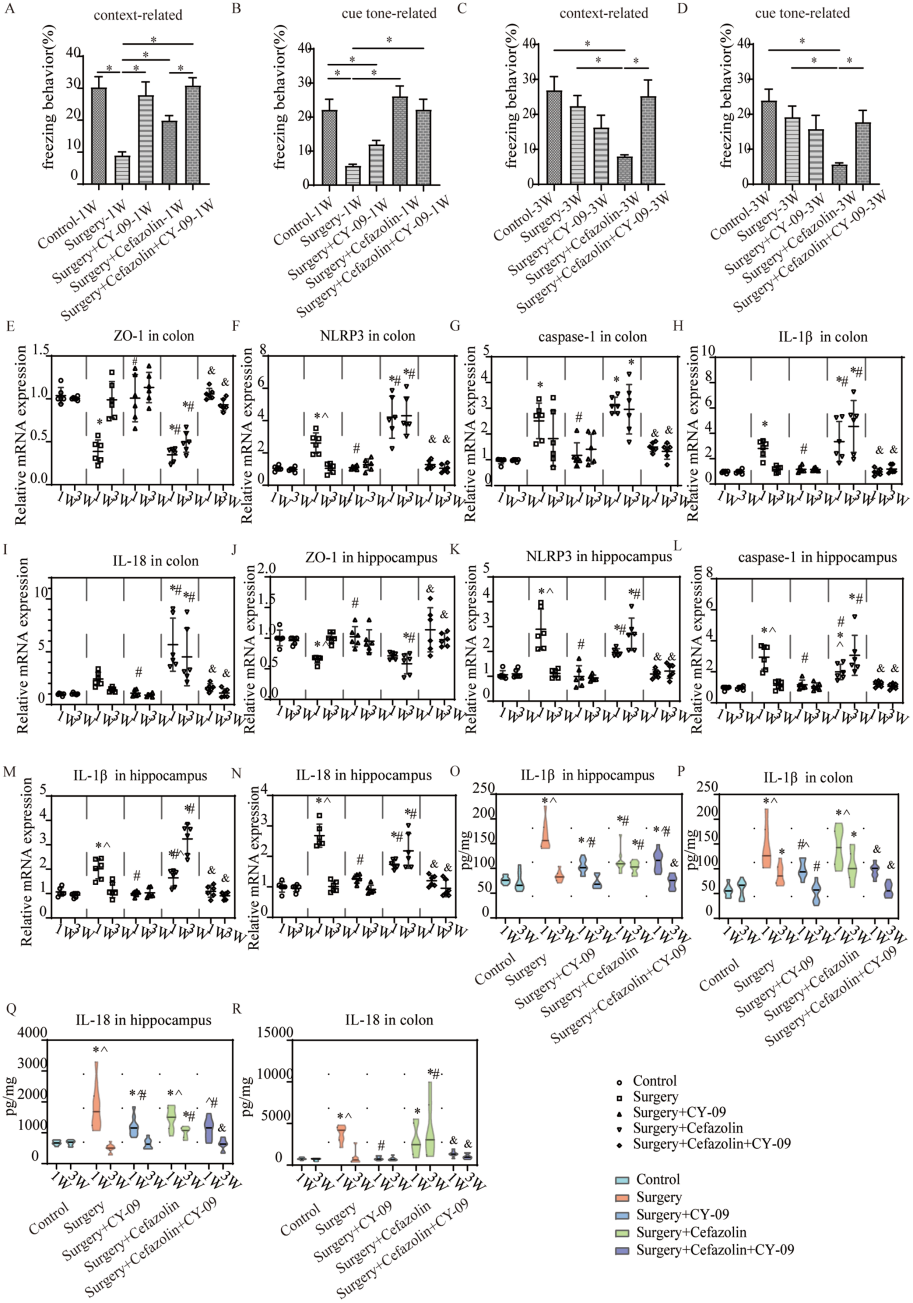
The CY-09 administration effectively improved perioperative cognitive dysfunction, inhibited perioperative inflammatory response, and protected barrier permeability in the colon and hippocampus. **(A–D)** CY-09 improved context-related freezing behavior (q = 6.952, *p* = 0.0001) decreased by surgery/anesthesia stress at 1 week. While at 3 weeks after surgery, CY-09 also improved context-related (q = 5.112, *p* = 0.0065) and cue tone-relatedFIGURE 9 (Continued)(Z = 3.222, *p* = 0.0127) freezing behavior compared with surgery + cefazolin group (*n* = 10, ANOVA with Tukey’s multiple comparisons tests, or Kruskal-Wallis test with Dunn’s multiple comparisons test when SDs are significantly different). **(E,J)** PCR: CY-09 could recover the decreasing level of ZO-1 in the colon (q = 10.11, *p* < 0.0001) and hippocampus (q = 4.802, *p* = 0.0177) caused by surgery/anesthesia stress at 1 week, while CY-09 could increase these decreases of ZO-1 in the colon (1 week, q = 11.54, *p* < 0.0001; 3 weeks, q = 7.640, *p* = 0.0001) and hippocampus (1 week, q = 5.501, *p* = 0.0054; 3 weeks, q = 7.391, *p* = 0.0002) both at 1 and 3 weeks after surgery compared with surgery + cefazolin group (*n* = 6, ANOVA with Tukey’s multiple comparisons test). **(F–N)** PCR: CY-09 decreased the increasing level of NLRP3, caspase-1, IL-1β, IL-18 in colon (NLRP3, q = 5.605, *p* = 0.0045; caspase-1, q = 7.923, *p* < 0.0001; IL-1β, q = 5.096, *p* = 0.0109; IL-18, q = 2.763, *p* = 0.3168) and hippocampus (NLRP3, q = 10.79, *p* < 0.0001; caspase-1, q = 9.790, *p* < 0.0001; IL-1β, q = 8.879, *p* < 0.0001; IL-18, q = 15.49, *p* < 0.0001) caused by surgery/anesthesia stress at 1 week, while CY-09 could decrease the increasing level of these factors both at 1 week (in the colon: NLRP3, q = 10.84, *p* < 0.0001; caspase-1, q = 9.618, *p* < 0.0001; IL-1β, q = 7.420, *p* = 0.0002; IL-18, q = 8.445, *p* < 0.0001; in hippocampus: NLRP3, q = 4.935, *p* = 0.0142; caspase-1, q = 4.310, *p* = 0.0392; IL-1β, q = 4.831, *p* = 0.0169; IL-18, q = 5.931, *p* = 0.0025) and 3 weeks (in colon: NLRP3, q = 13.94, *p* < 0.0001; caspase-1, q = 5.649, *p* = 0.0042; IL-1β, q = 8.815, *p* < 0.0001; IL-18, q = 6.761, *p* = 0.0006; in hippocampus: NLRP3, q = 10.62, *p* < 0.0001; caspase-1, q = 8.074, *p* < 0.0001; IL-1β, q = 17.28, *p* < 0.0001; IL-18, q = 9.894, *p* < 0.0001) after surgery compared with surgery + cefazolin group (*n* = 6, ANOVA with Tukey’s multiple comparisons test). **(O–R)** ELISA: CY-09 decreased the increasing level of IL-1β, IL-18 in the colon (IL-1β, q = 4.154, *p* = 0.0460; IL-18, q = 8.440, *p* < 0.0001) and hippocampus (IL-1β, q = 9.971, *p* < 0.0001; IL-18, q = 4.842, *p* = 0.0111) caused by surgery/anesthesia stress at 1 week, while CY-09 could decrease the increasing level of IL-1β (1 week, q = 4.451, *p* = 0.0282; 3 weeks, q = 7.393, *p* < 0.0001), IL-18 (1 week, q = 4.362, *p* = 0.0327; 3 weeks, q = 5.364, *p* = 0.0056) in the colon at 1 and 3 weeks and IL-1β, IL-18 in the hippocampus (IL-1β, q = 7.063, *p* < 0.0001; IL-18, q = 8.472, *p* < 0.0001) only at 3 weeks (*n* = 6, ANOVA with Tukey’s multiple comparisons test). ^*^Compared with Grp CTL, *p* < 0.05; ^#^compared with Grp S, *p* < 0.05; ^&^compared with Grp SC, *p* < 0.05; ^^^compared with 3 weeks, *p* < 0.05.

Intraperitoneal CY-09 treatment, a potent, selective inhibitor of NLRP3 inflammasome activation, could be a significant factor in increasing ZO-1 and decreasing NLRP3/caspase-1 and IL-1β, IL-18 expression in the colon and hippocampus 1 week after surgery in Grp S, and 1 and 3 weeks after surgery in Grp SC (Figure 9). These findings indicate that CY-09 inhibits the inflammatory response following gut dysbiosis through a mechanism distinct from probiotics, which influence dysbiosis regulation. Furthermore, CY-09 could significantly improve the context-related and cue tone-related cognitive deficits caused by surgery/anesthesia stress and surgery/anesthesia stress combined with cefazolin 1 week after surgery (Figures 9A,B). Moreover, CY-09 improved the cognitive deficits induced by surgery/anesthesia stress combined with cefazolin 3 weeks after surgery (Figures 9C,D).

### Surgery/anesthesia stress enhanced the function of microbiota for peptidoglycan biosynthesis, an effect suppressed by probiotics

**Figure 10:**
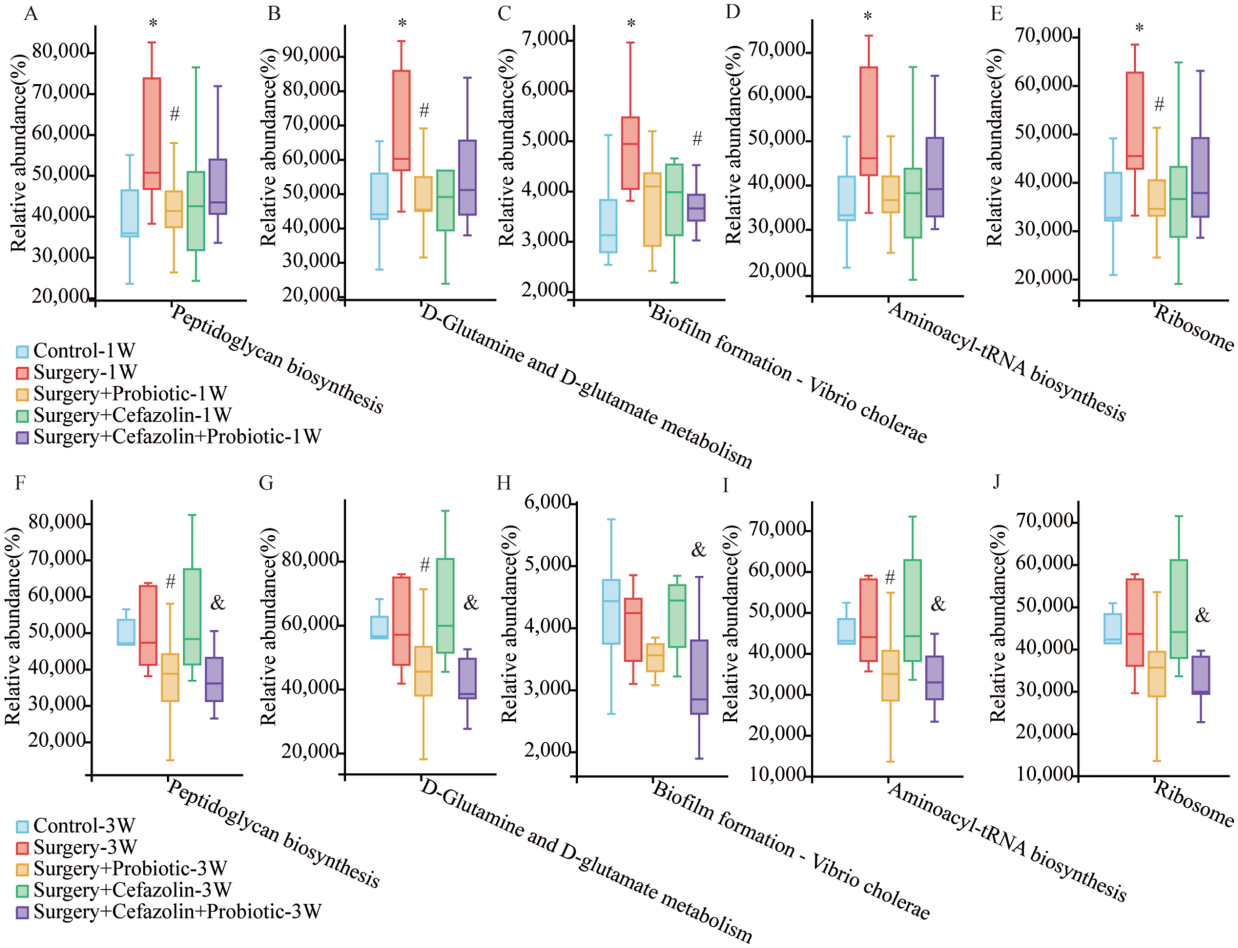
PICRUSt2 functional predictive analysis based on the Greengene database. **(A–E)** The relative abundance of Peptidoglycan biosynthesis, D-Glutamine and D-glutamate metabolism, Biofilm formation—Vibrio cholerae, Aminoacyl-tRNA biosynthesis, and Ribosome at postoperative 1 week among Grp CTL, Grp S, Grp SP, Grp SC, Grp SCP. **(F–J)** The relative abundance of Peptidoglycan biosynthesis, D-Glutamine and D-glutamate metabolism, Biofilm formation—Vibrio cholerae, Aminoacyl-tRNA biosynthesis, and Ribosome at postoperative 3 weeks among Grp CTL, Grp S, Grp SP, Grp SC, Grp SCP. Grp, group; CTL, control; S, surgery; SC, surgery combined with cefazolin; SP, surgery combined with probiotics; SCP, surgery combined with cefazolin and probiotics. ^*^Compared with Grp CTL, *p* < 0.05; ^#^compared with Grp S, *p* < 0.05; ^&^compared with Grp SC, *p* < 0.05.

It has been reported that peptidoglycan on the cytoderm of Gram-positive bacteria can lead to NLRP3 inflammasome activation and the secretion of IL-1β by toll-like receptor 2 (TLR2; Liang et al., 2013; Miller et al., 2021). What functions of the intestinal microbiome are adjusted by dysbiosis? Surgery/anesthesia stress enhanced the function of the microbiota for peptidoglycan biosynthesis, D-glutamine, and D-glutamate metabolism, biofilm formation of vibrio cholerae, aminoacyl-tRNA biosynthesis, and ribosome at 1 week postoperatively, which may be related to the change in the G^+^/G^−^ ratio as determined by additional analysis on PICRUSt2 using the green-gene database (Figure 10). It should be noted that probiotics could suppress these functions. However, these alterations in function generated by surgery/anesthesia stress exhibited a recovery pattern. At 3 weeks postoperatively, probiotics could suppress the five boosting activities generated by cefazolin.

## Discussion

Cefazolin, a cephalosporin of the first generation, has been widely utilized. As with other antibiotics, the antibacterial activity can profoundly alter the intestinal microbiota (Fröhlich et al., 2016). Our study showed that cefazolin could decrease the richness and evenness of the microbiome. After therapy suspension, the alpha diversity was gradually restored, but the richness restoration lagged. Recently, the contribution of surgery to gut dysbiosis has been documented (Ohigashi et al., 2013; Aardema et al., 2019). Surgery on peripheral tissues and organs induces systemic inflammation and can significantly disturb the intestinal microbiota (Ohigashi et al., 2013). Different from cefazolin-induced dysbacteriosis, we discovered that surgery/anesthesia stress decreased the number of bacteria. These results indicated that both cefazolin and surgery/anesthesia stress could affect the balance of gut microbiota, but they had their characteristics.

Gut microbiota has also been regarded as mind-altering microorganisms (Cryan and Dinan, 2012). It has an impact on the blood–brain barrier (BBB) permeability (Braniste et al., 2014), normal metabolism (Morrison and Preston, 2016), and neurodegeneration (Hu et al., 2016; Cho et al., 2021; Yang et al., 2021). The microbiome is crucial in a healthy brain and cognition (Liu et al., 2019; Cuervo-Zanatta et al., 2021). An altered composition of the microbiota can lead to sustained inflammation and further neuroinflammation (Sampson et al., 2016; Wong et al., 2016; Tse, 2017). Neuroinflammation is one of the mechanisms associated with cognitive dysfunction (Daulatzai, 2014; Giridharan et al., 2020). These results indicate that an acute modification in gut microbiota has a substantial association with systemic inflammation and further cognitive function alterations. After measuring gut dysbacteria after cefazolin and surgery/anesthesia stress, we found there is closely relevance between both these two types of gut dysbacteria and cognitive deficits. Dysbacteria is accompanied by colonic inflammation activation, and then high IL-1β and IL-18 levels may transfer through BBB by gut-brain axis to induce central neuroinflammation. Therefore, cognition will be impaired.

However, antibiotics’ anti-inflammatory properties can relieve neuroinflammation and cognitive impairment. Preoperative oral administration of antibiotics improved postoperative learning and memory in mice (Jiang et al., 2019). It has been demonstrated that minocycline mitigates isoflurane-induced cognitive impairment in aged rats (Li et al., 2013). According to reports, cefazolin is a common gamma-chain cytokine inhibitor with anti-inflammatory properties (Zyzynska-Granica et al., 2020). Our research group has recently demonstrated that cefazolin can inhibit lipopolysaccharide-induced inflammation (Liang et al., 2018). This study found that perioperative intraperitoneal injection of cefazolin for 6 days effectively attenuated the actions of proinflammatory cytokines in the hippocampus and ameliorated short-term postoperative cognitive deficits. These results suggest that the direct anti-inflammatory effects of cefazolin can inhibit surgery/anesthesia-induced central nervous IR and improve short-term postoperative cognition.

Surprisingly, we discovered that 3 weeks after surgery, cefazolin decreased the freezing behavior of mice, indicating a deleterious effect of cefazolin on long-term postoperative neurocognition. Consistent with this suggestion, IL-1β and IL-18 levels were increased by cefazolin in the hippocampus 3 weeks after surgery. Moreover, there was an increase in IL-1β and IL-18 in the colons of mice treated with surgery and cefazolin. Perioperative cefazolin treatment almost eliminated the 16S rRNA expression in the colon, suggesting a severe decrease in gut microbiota (Liang et al., 2018). Furthermore, in this study, we investigated the diversity and composition of gut bacteria and found that cefazolin could inhibit the restoration of gut dysbacteria caused by surgery/anesthesia. The perioperative administration of cefazolin altered not only the number but also the composition of microorganisms. A high level of IL-1β in the colon appeared persistently after surgery. Under these circumstances, the delayed restoration of cefazolin-induced gut dysbacteria resulted in colonic and hippocampus inflammation and cognitive impairment. These findings suggest that cefazolin’s anti-microbiota activity can inhibit the regrowth of postoperative dysbacteria and affect long-term postoperative cognition. Therefore, we consider that perioperative cefazolin has a double-edged effect on postoperative cognition.

Exogenous probiotics and prebiotics may effectively correct intestinal microbiota imbalance and reduce colonic inflammation (Jakubczyk et al., 2020). Several studies have elucidated this direct effect on the behavior of microorganisms. Lactobacillus inhibits inflammation and oxidation stress in mice with cerebral ischemia (Wanchao et al., 2018; Xu et al., 2018). Orally supplementary probiotics 2 weeks before surgery could correct the dysbacteriosis and further attenuate IR and cognitive disorders (Yu et al., 2019). Additionally, FOS, as one of the prebiotics, could exert an anti-AD effect in mice, and the involved mechanisms were attributed to the gut microbiota-GLP-1/GLP-1R pathway (Sun et al., 2019). Our study found that perioperative FOS + probiotics treatment is an efficient method to correct gut dysbacteria. NLRP3 inflammasome activation and IR in the colon were suppressed considerably, although enteric epithelial barrier (EEB) and BBB were protected from inflammatory stimulation. Therefore, central neuroinflammation and cognitive dysfunction were ameliorated by FOS + probiotics therapy. According to a recent population-based study, perioperative probiotics administration effectively reduced PND morbidity and the degree of postoperative systematic inflammation (Wang P. et al., 2021), which is compatible with our findings. These results suggest that perioperative administration of FOS + probiotics is an effective and safe method to prevent PND.

However, because probiotics are sensitive to antibiotics, supplementing them exogenously during antibiotic therapy might be challenging. Our results indicate that probiotics cannot change the diversity and cognition 1 week after surgery in mice who had surgery and cefazolin. However, postoperatively at 3 weeks, probiotics improved the dysbacteriosis and long-term cognitive dysfunction. Nonetheless, with the cefazolin suspension, sustainable probiotics supplement does not bring gut microbiota back to normal. It may be because probiotics may prolong the reconstruction of the local microbiome after antibiotic treatment, a phenomenon known as probiotic-related gut dysbiosis (Suez et al., 2018). In this circumstance, we also found a similarly increasing *Akkermansia* and reducing alpha diversity as Suez’s research. This finding indicated that in the context of surgery, the interaction between probiotics and antibiotics is equal to that without surgery.

Additionally, a post-antibiotic non-ecological niche condition can improve the microbiome recolonization by probiotics, enhancing its advantages (Suez et al., 2018). We deem that dysbacteria in mice of Grp SCP at 3 weeks can be considered a type of post-antibiotic probiotic-related gut dysbiosis. Although alpha diversity is low, probiotics’ other purpose is likely to increase it. Hence, the deficiency of microbiome recolonization can be offset. *Akkermansia* is regarded as a potential emerging probiotic (Zhang et al., 2019; Zhai Q. et al., 2019), which can be used for repairing EEB (Hiippala et al., 2018), attenuating IR (Zhai R. et al., 2019) and improving insulin resistance (Depommier et al., 2019). Furthermore, *Akkermansia* influences hippocampal development and cognitive function (Yang et al., 2019; Ou et al., 2020). Therefore, we conclude that the enhancement of long-term cognition in Grp SCP mice can be partially ascribed to the increased prevalence of *Akkermansia* but not to exogenously administered probiotic administration.

Unexpectedly, our findings revealed a negative connection between cognitive change and the G^+^/G^−^ ratio. Both probiotics and cefazolin can adjust G^+^/G^−^ ratio by inhibiting the increasing abundance of *Streptococcus*, thus remodeling cognitive deficits caused by surgery/anesthesia stress. Regarding cefazolin-induced long-term PND, the modifying effects of probiotics on the G^+^/G^−^ ratio are mostly attributable to the decrease in Streptococcus and the increase in *Akkermansia*. Additionally, peptidoglycan on the cytoderm of gram-positive bacteria can lead to the NLRP3 inflammasome activation and the secretion of IL-1β by TLR2 (Liang et al., 2013; Miller et al., 2021). The PICRUSt2 functional predictive analysis found that peptidoglycan biosynthesis was significantly enhanced by surgery/anesthesia stress, which was consistent with an increasing G^+^/G^−^ ratio. NLRP3 has been reportedly involved with inflammatory bowel disease (IBD), Alzheimer’s disease, and PND. In conclusion, we hypothesize that a high ratio of G^+^/G^−^ may promote NLRP3 pathway activation by stimulating TLR2 through peptidoglycan activities, leading to cognitive problems that can be treated with probiotics.

Our results revealed that CY-09 inhibited NLRP3 and its downstream activation and thus reduced inflammation and cognitive impairment. CY-09’s suppression of the NLRP3/caspase-1 pathway can maintain the integrity of the EEB and BBB and enhance cognition following intestinal dysbacteria. Interestingly, cognition at 1 week in mice of Grp SC can be significantly improved by CY-09 but not probiotics. These results suggest that CY-09 may come into play after dysbiosis has occurred.

We designed this study to simulate clinical settings. Although much comparative research relies on the oral administration of antibiotics, intraperitoneal injection of cefazolin is more likely to replicate intravenous treatment. The administration of cefazolin orally has a more direct effect in eliminating the gut microbiota. However, perioperative antibiotics therapy is more inclined to be systemic. Moreover, this delivery can be essential to separate antibiotic and probiotic actions, helping to avoid interactions and disturbances between these two treatments in the gastrointestinal tract. Since female mice experience menstruation, gut microbiome and behavior tests may be impacted; hence we only use male mice for research. Even though advanced age is a major factor in PND risk, and it seems like the old mice might be more suitable for this study, we chose only young mice for economic limitations. We discovered a considerable difference in the cognitive abilities of young mice. Given the increasing prevalence of PND in older mice, we assumed that our results were, to some extent, comparable to those of older animals.

Our findings have significant implications. The anti-inflammation and anti-microorganism actions of cefazolin have a double effect on postoperative cognitive disorders. The differential effect is time-based, which presents an anti-inflammatory effect that can improve short-term cognition. In contrast, the anti-microorganism effect can prolong long-term cognitive impairment. Effective and efficient probiotics can facilitate the recolonization of microbiota and improve cognition. Probiotic advantages do not always show during antibiotic therapy, but the G^+^/G^−^ ratio is highly relevant to cognitive change. *Streptococcus* and *Akkermansia* could be two types of cognition-dependent flora.

Our study has several limitations. First, 16S rRNA is restricted to the genus and cannot be utilized for species analysis. It prohibits us from investigating further the effect of specific species on memory. Second, after determining the relationship between the G^+^/G^−^ ratio and cognitive function, we deduced that PGN-induced inflammatory activation would impact cognition based on existing published research. Further studies will be performed soon to identify the effects of PGN on cognitive deficits. Third, based on our previous study, increasing IL-1β levels in serum could be detected 24 h after surgery but not persistently after 1 week (Liang et al., 2018). Therefore, in our study, the IL-1β level in serum is not regarded as a reliable indicator. The elevated level of proinflammatory cytokines in the colon and hippocampus, which persisted one or 3 weeks after surgery, may have been a delayed response or unrelated to serum IL-1β levels as a mediator. Due to the complexity and susceptibility of intestinal microbiota, our study has a limitation for the definition of cognition-dependence bacteria.

## Conclusion

Perioperative probiotics administration can repair the gut microbiota and enhance PND, which may inhibit the NLRP3/caspase-1 pathway. The double effect on surgery-induced memory deficits of cefazolin shows a time-based differential. The findings indicate that the perioperative gut microbiome balance, particularly the G^+^/G^−^ ratio restored by probiotics, may be closely related to postoperative cognitive disorders.

## Significance statement

Several studies examine the relationship between gut microbiota and cognitive function. The perioperative inflammatory response may result in gut dysbacteria, further linked with perioperative neurocognitive disorder. This study examines the effects of probiotics, cefazolin, or both on various perioperative gut dysbacteria. We found that perioperative cefazolin has a double-edged effect on postoperative cognition, which can improve short-term cognitive dysfunction but decrease long-term cognitive dysfunction. Additionally, perioperative probiotic administration can ameliorate perioperative neurocognitive disorder by rebuilding the gut microbiota, which may inhibit the NLRP3/caspase-1 pathway. Our findings indicate that the perioperative gut microbiome balance, particularly the G^+^/G^−^ ratio, may be closely related to the perioperative neurocognitive disorder. *Streptococcus* and *Akkermansia* may represent two types of cognition-based flora.

## Data availability statement

The datasets presented in this study can be found in online repositories (https://submit.ncbi.nlm.nih.gov/subs/sra/SUB13029354/overview).

## Ethics statement

The animal study was reviewed and approved by Animal Ethics Committee of West China Hospital of Sichuan University.

## Author contributions

PL conceived the project and performed the final manuscript. TZ, CZ, HH, and PL designed the study. XW did the initial data analysis. TZ performed the experiments, did the final data analysis, and drafted the initial manuscript. All authors contributed to the article and approved the submitted version.

## Funding

This work was supported by the grants no. 81600918 (to PL) and no. 81974164 (to CZ) from National Natural Science Foundation of China (Beijing, China), and no.23NSFSC4876(to TYZ) from Natural Science Foundation of Sichuan, and Disciplinary Construction Innovation Team Foundation of Chengdu Medical College: CMC-XK-2101(to TYZ).

## Conflict of interest

The authors declare that the research was conducted in the absence of any commercial or financial relationships that could be construed as a potential conflict of interest.

## Publisher’s note

All claims expressed in this article are solely those of the authors and do not necessarily represent those of their affiliated organizations, or those of the publisher, the editors and the reviewers. Any product that may be evaluated in this article, or claim that may be made by its manufacturer, is not guaranteed or endorsed by the publisher.

## Footnotes

^1^ http://www.omicsmart.com

## Abbreviations

MGBA: Microbiota-gut-brain axis
PPIs: Proton pump inhibitors
FC: Fear conditioning
IL-1β: Interleukin-1β
IL-18: Interleukin-18
TJP/ZO-1: Tight junction protein
LPS: Lipopolysaccharide
NS: Normal saline
PND: Perioperative neurocognitive disorder
CFU: Colony Forming Units
AGE: Agarose gel electrophoresis
G^+^: Gram-positive bacteria
G^−^: Gram-negative bacteria
TLR2: Toll-like receptor 2
